# In vitro assessment of Lipiodol-targeted radiotherapy for liver and colorectal cancer cell lines

**DOI:** 10.1054/bjoc.1999.0950

**Published:** 2000-01-18

**Authors:** S Ho1, W Y Lau, W T Leung

**Affiliations:** Department of Clinical Oncology and Surgery, The Chinese University of Hong Kong, Prince of Wales Hospital, Shatin, Hong Kong


*British Journal of Cancer* (2000) **82**(2), 497–498
© 2000 Cancer Research Campaign
Article no. bjoc.1999.0950

Letter to the Editor

### In vitro assessment of Lipiodol-targeted radiotherapy for liver and colorectal cancer cell lines

**Sir**

We read with great interest the article by Al-Mufti et al (1999) (*Br J Cancer* **79**: 1665–1671). However, we found the concept on which their study design was based needs to be clarified. The cytotoxic action of a chemotherapeutic drug on cancer cells is different from that of a therapeutic radiopharmaceutical. For a chemotherapeutic drug such as a methylating agent to produce its cytotoxicity, its molecules need to enter the cancer cells before it can act on their DNA. That is why P-glycoprotein, which causes efflux of the drug molecule out of the cells, leads to drug resistance. On the other hand, the radiation-emiting isotope contained in a radiopharmaceutical produces its cytotoxic effect by bombarding the DNA of the cancer cells with beta particles or depositing radiation energy to the cells in some other manner. This can be achieved as long as the cancer cells are within the range at which the radiation can penetrate.

For iodine-131 ($^{131}$I) the beta-particle it emits carries the majority of the radiation energy. The mean penetration of its beta particle in soft tissues is 0.4 mm, which is equivalent to 16–160 cell diameters. By the inverse-square law, the closer the radiopharmaceutical molecule to the cancer cell, the greater will be the amount of energy deposited, but it is not necessary for the radiopharmaceutical molecules to enter the cancer cells before cytotoxicity can be produced. In this study, the authors wanted to demonstrate that $^{131}$I produces its preferential cytotoxic effects only when it is inside the malignant cell (intracellular radiotherapy, in the words of the authors) and Lipiodol was essential for transporting the radionuclide into the cell. They tried to show that the Lipiodol cannot enter the cancer cells in the absence of $^{131}$I and so produces no cytotoxic effects. The same happens for $^{131}$I alone without Lipiodol. As stated by the authors, the iodine ($^{127}$I) content of Lipiodol is 38–40% w/v (Al-Mufti et al, 1999), whereas < 4.2 μg of sodium iodide is present in every 25 mCi of $^{131}$I solution we used to label 1 ml of Lipiodol (Lau et al, 1999). Therefore the percentage of $^{127}$I in Lipiodol being exchanged by $^{131}$I during the radiolabelling is negligible. The conversion of $^{127}$I-Lipiodol to $^{131}$I-Lipiodol should not have any change in the physiochemical properties and therefore the cells should have recognized both the non-radioactive and the radioactive Lipiodol as identical. Nevertheless, the authors reported very different electron micrography patterns of the two kinds of Lipiodol in both the malignant and benign cells (Al-Mufti et al, 1999). Cold Lipiodol appeared in the form of cytoplasmic membrane-bound vesicles, whereas $^{131}$I-lipiodol was detected inside dead cancer cells and viable benign cells which are claimed to be healthy by the authors.

Lipiodol (density 1.28 g ml$^{-1}$) is denser than aqueous culture medium and the two liquids are immiscible. From our experience, even in the presence of Urografin as an emulsifying agent, the emulsion state could not be prolonged for more than 1 h (unless it was continuously agitated) and the Lipiodol still separated out and settled at the bottom on standing. Hence their experimental observations might be interpreted as follows. On standing inside the incubator for over 6 h, the $^{131}$I-Lipiodol component of the emulsion has separated and sunk. It came into intimate contact with the monolayer of cells at the bottom of the well. The local radioactivity concentrations in the vicinity of the cells were 1.0, 2.0 and 4.0 μCi of $^{131}$I in 4 μl of $^{131}$I-Lipiodol (equivalent to 250, 500 and 1000 μCi ml$^{-1}$ for low, medium and high dose). Thus a high radiation dose was delivered to the cells. When the cells are killed or damaged by the radiation, the cells lost their integrity and the cell membrane became permeable to the passage of $^{131}$I-Lipiodol which ended up inside the cells. The endothelial cells should have a lower population of dividing cells which are more susceptible to radiation damage and so the effects on the benign cells were seen to be sub-lethal while the malignant cells were killed. However, the integrity of the endothelial cell might still have been affected in some way and so the $^{131}$I-Lipiodol could gain its passage into some of these benign cells.

Such radiation effects were not observed with aqueous NaI ($^{131}$I) solution. The aqueous NaI ($^{131}$I) solution, being completely miscible with the culture medium, will be distributed uniformly throughout the 100 μl of medium and therefore the effective radioactivity concentrations in the vicinity of the cells was really 40 μCi ml$^{-1}$. So even the low dose $^{131}$I-Lipiodol was providing a 6.25 times higher local radioactivity concentration of $^{131}$I than in the aqueous NaI ($^{131}$I) solution. It is therefore logical for the authors to observe no cytotoxic effect in the NaI ($^{131}$I) solution despite the total radioactivity in the well was four times that of the low dose $^{131}$I-Lipiodol. We can assure the authors of a similar cell killing effect as observed with $^{131}$I-Lipiodol if they try to increase the NaI ($^{131}$I) concentration to 250 μCi ml$^{-1}$.

From our own experience in treating 26 patients with hepatocellular carcinoma using $^{131}$I-Lipiodol (Leung et al, 1994) and the results of the other 12 clinical series of hepatic cancer treated with the same agent that have recently been reviewed by us (Ho et al, 1998) we agree with the authors that the response is highly variable and may partly depend on local pharmacokinetics. The other important factor of course is the radio-sensitivity of the tumour cells. As we have pointed out in our review article (Ho et al, 1998) for a lesion to be completely destroyed by $^{131}$I-Lipiodol, it needs to uptake the agent in such a way that every cancer cell lies within 2.4 mm from the radioactive oil, which is the maximum penetration of the beta-radiation from $^{131}$I. $^{131}$I-Lipiodol could hardly concentrate in hypovascular or necrotic tumours and therefore their responses are poor.

In conclusion, $^{131}$I-Lipiodol bound to cytoplasmic membrane of the cancer cell as vesicles of lipids is already at a sufficiently close distance to kill the cancer cells. It is not necessary for the $^{131}$I-Lipiodol molecules to enter the cancer cell before it can produce its cytotoxicity.

*S Ho[1], WY Lau[2] and WT Leung[1]*
*Departments of [1]Clinical Oncology and [2]Surgery,*
*The Chinese University of Hong Kong,*
*Prince of Wales Hospital,*
*Shatin, Hong Kong*

### REFERENCES

Al-Mufti R, Pedley RB, Marshall D, Begent RH, Hilson A, Winslet MC and Hobbs KEF (1999) In vitro assessment of Lipiodol-targeted radiotherapy for liver and colorectal cancer cell lines. *Br J Cancer* **79**: 1665–1671

Ho S, Lau WY, Leung TWT and Johnson PJ (1998) Internal radiation therapy for patients with primary or metastatic hepatic cancer. A review. *Cancer* **83:** 1894–1907

Lau WY, Leung TWT, Ho SKW, Chan M, Machin D, Lau J, Chan ATC, Yeo W, Mok TSK, Yu SCH, Leung N and Johnson PJ (1999) Adjuvant intra-arterial iodine-131-labelled lipiodol for resectable hepatocellular carcinoma – a prospective randomized trial. *Lancet* **353:** 797–801

Leung WT, Lau WY, Ho S, Chan M, Leung N, Lin J, Ho KC, Metreweli C, Johnson PJ and Li AKC (1994) Selective internal radiation therapy with intraarterial $^{131}$Iodine-Lipiodol in inoperable hepatocellular carcinoma. *J Nucl Med* **35:** 1313–1318

*© 2000 Cancer Research Campaign*

